# Subdiffusion in Membrane Permeation of Small Molecules

**DOI:** 10.1038/srep35913

**Published:** 2016-11-02

**Authors:** Christophe Chipot, Jeffrey Comer

**Affiliations:** 1 Laboratoire International Associé Centre National de la Recherche Scientifique et University of Illinois at Urbana-Champaign, Unité Mixte de Recherche n°7565, Université de Lorraine, B.P. 70239, 54506, Vandœuvre-lès-Nancy cedex, France; 2 Theoretical and Computational Biophysics Group, Beckman Institute for Advanced Science and Technology, University of Illinois at Urbana-Champaign, 405 North Mathews Avenue, Urbana, Illinois 61801, USA; 3 Department of Physics, University of Illinois at Urbana-Champaign, 1110 West Green Street, Urbana, Illinois 61801, USA; 4 Institute of Computational Comparative Medicine, Nanotechnology Innovation Center of Kansas State, Department of Anatomy and Physiology, 1800 Denison Ave, Kansas State University, Manhattan, Kansas 66506, USA

## Abstract

Within the solubility–diffusion model of passive membrane permeation of small molecules, translocation of the permeant across the biological membrane is traditionally assumed to obey the Smoluchowski diffusion equation, which is germane for classical diffusion on an inhomogeneous free-energy and diffusivity landscape. This equation, however, cannot accommodate subdiffusive regimes, which have long been recognized in lipid bilayer dynamics, notably in the lateral diffusion of individual lipids. Through extensive biased and unbiased molecular dynamics simulations, we show that one-dimensional translocation of methanol across a pure lipid membrane remains subdiffusive on timescales approaching typical permeation times. Analysis of permeant motion within the lipid bilayer reveals that, in the absence of a net force, the mean squared displacement depends on time as *t*^0.7^, in stark contrast with the conventional model, which assumes a strictly linear dependence. We further show that an alternate model using a fractional-derivative generalization of the Smoluchowski equation provides a rigorous framework for describing the motion of the permeant molecule on the pico- to nanosecond timescale. The observed subdiffusive behavior appears to emerge from a crossover between small-scale rattling of the permeant around its present position in the membrane and larger-scale displacements precipitated by the formation of transient voids.

In the search of novel therapeutic agents, many chemical compounds able to bind a given target with very high affinity are eventually discarded on account of their cytotoxicity, their propensity to associate to potassium channel hERG^1^, or their poor bioavailability. Predicting these properties at an early stage of drug discovery, upstream from costly organic syntheses and clinical trials, is, therefore, desirable. One possible avenue to address high drug-attrition rates^2^ consists in quantifying the ability of the substrate to spontaneously traverse lipid membranes, for instance, in the gastrointestinal tract, and reach the targeted protein in an adequate amount. A consistent theoretical model of the lipid membrane permeation process is, therefore, essential for linking the physicochemical properties of drug candidates to their adsorption and distribution. In pharmaceutical settings, this quantity is determined using models like the parallel artificial membrane permeability assay^3^, or the colorectal carcinoma cell-based assay^4^, which consists of a heterogenous lipid environment. Experiments often resort to an indirect measurement of the permeability, employing a micropipette-aspiration technique^5^, wherein the mechanical properties of a lipid vesicle immersed in an aqueous solution of the permeant are determined. The permeability is then inferred from the apparent variation of the surface area of the vesicle induced by the flux of substrates therein. A convenient framework for understanding permeation has been provided by the inhomogeneous solubility-diffusion model^6^, which relates the resistance to permeation in the direction, *z*, normal to the membrane to the position-dependent diffusivity of the substrate, *D*(*z*), and the potential of mean force (PMF), or one-dimensional free-energy profile, *w*(*z*), underlying its translocation from the bulk aqueous phase to the interior of the lipid environment,


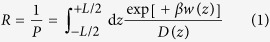


where *w*(*z*) is defined to be zero for *z* in bulk water, *β* = 1/*k*_B_*T*, and *k*_B_ is the Boltzmann constant and *T*, the temperature. The bounds of the integral are chosen to span the entire membrane, extending from the bulk water on one side of the lipid environment to the bulk water on the other side. It is apparent from Equation 1 that accurate calculation of the permeability depends on how well the free-energy change for moving the permeant from the aqueous medium into the lipid bilayer can be reproduced, and on our aptitude to describe appropriately diffusive kinetics of the substrate within the membrane.

Equation 1 can be derived^7^ from the Smoluchowski diffusion equation^8^,^9^, which describes diffusion of a particle under the influence of a deterministic force (e.g. on a nonuniform free-energy landscape):


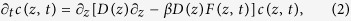


where *c*(*z*, *t*) is the concentration of the permeant and *F*(*z*, *t*) is the deterministic force, obeying *F*(*z*, *t*) = −∂_*z*_*w*(*z*) for conservative systems.

Equation 1 inherently describes a Markovian process that is also local in space. However, a distinct form of diffusive motion, referred to as subdiffusion^10^ and characterized by long-range correlations in time or space^11^, has been recognized in many biological systems^12^, including diffusion in crowded cytoplasm^13^, internal dynamics of proteins^14^, and gating of ion channels^15^. Note that the physical origin of subdiffusion, and even whether it is truly present, remains controversial for some of these systems^16^. Although subdiffusion is known to extend to arbitrarily long timescales for some systems, such as an obstructed medium at the percolation threshold, transient subdiffusion over restricted timescale may be more common^17^. One system for which transient subdiffusion is well known is the lateral diffusion of lipids in the phospholipid bilayer through which permeation occurs^18^,^19^. For times less than about 100 fs, inertia dominates and motion of the lipids is ballistic, with mean squared displacements (MSD) of 〈*x*^2^〉 ~ *t*^2^, while on timescales beyond about 10 ns, the aggregate effect of a large number of interactions yields a classical random walk with 〈*x*^2^〉 ~ *t*. However, between these two timescales, spanning about five orders of magnitude, the lipids move in a manner that is asymptotically slower than any classical diffusion process. This is subdiffusion, where 〈*x*^2^〉 ~ *t*^*α*^, with 0 < *α* < 1. Recent experimental work suggests that subdiffusion is relevant on timescales reaching many seconds in multicomponent membranes^20^.

The lateral diffusion of lipids in a pure bilayer is a particularly simple case because the free energy and diffusivity are independent of the position *x* by symmetry. For nonuniform free-energy landscapes and diffusivities, neither a linear nor power-law dependence of the MSD is to be expected, making interpretation of the diffusive regime more complicated. Below, we circumvent this complication by performing simulations in which the free-energy landscape is artificially made uniform by applying an external force, or by using lateral diffusion as a proxy for diffusion along *z*. However, note that the final test of our model considers unbiased diffusion on the rugged natural free-energy landscape.

Given the subdiffusive behavior of lipids in membranes on the timescale described above, an obvious question is whether small molecules in such an environment might also display some type of anomalous diffusion on similar timescales and whether this behavior might be relevant for permeation. However, most, if not all, theoretical descriptions of permeation up until now have assumed classical diffusion in their analysis of permeant motion. Here, using molecular dynamics, we carefully dissect the motion of methanol within a pure lipid bilayer with the aim of developing a rigorous theoretical framework for the description of permeation.

In some cases, observed anomalous diffusion is the result of projecting a multi-dimensional diffusion process onto a single variable, in which case an appropriate choice of a multi-dimensional space will remove the anomalous behavior. For instance, it has been shown that the correlation time of motion of a permeant transverse to a lipid bilayer can be significantly reduced by considering additional variables, such as atomic coordination numbers^21^. However, diffusion in such an abstract space becomes difficult to interpret and choosing the appropriate multi-dimensional space may not be straightforward. Furthermore, these additional variables may not be accessible to experiment. Here, we focus on developing a consistent model of effective diffusion along a single one-dimensional coordinate.

## Results and Discussion

### Free-energy profile

The exponential dependence of the permeability on *w*(*z*) in Equation 1 emphasizes the paramount importance of accurately reproducing the underlying free-energy change. For instance, errors as small as 1.4 kcal/mol are sufficient to bias the permeability by a factor of ten. In the context of solute permeation through lipid bilayers, several different approaches for rigorously computing free-energy profiles from molecular simulations have been used with comparable results, including constrained molecular dynamics^6^,^22^,^23^, umbrella sampling^24^,^25^, metadynamics^21^, bias-exchange metadynamics^26^, the oscillating forward-reverse method^27^, the adaptive biasing force (ABF) algorithm^28^,^29^ and multiple-walker ABF^25^. Two comprehensive reviews on the calculation of free energies for lipid bilayer permeation were recently provided by Neale and Pomès^30^ and Shinoda^31^. Using ABF, we calculated the free-energy profile for permeation of methanol through a palmitoyl-oleoyl-phosphatidylcholine (POPC) bilayer at a temperature of 308 K over the range of −45 ≤ *z* ≤ 45 Å, for a total of 3.6 *μ*s of simulated time. Figure S1 of the Supporting Information (SI) suggests that this time is more than sufficient to obtain a well-converged PMF. Given the inherent symmetry of the pure POPC bilayer, one can symmetrize this profile to yield an improved estimate of *w*(*z*). As detailed in Methods, we obtain a statistical uncertainty of roughly 0.2 kcal/mol at the center of the membrane, which contributes to a relative uncertainty in the permeability (applying Equation 1) of roughly 40%, which is sufficiently low for the purposes of this work.

**Figure 1:**
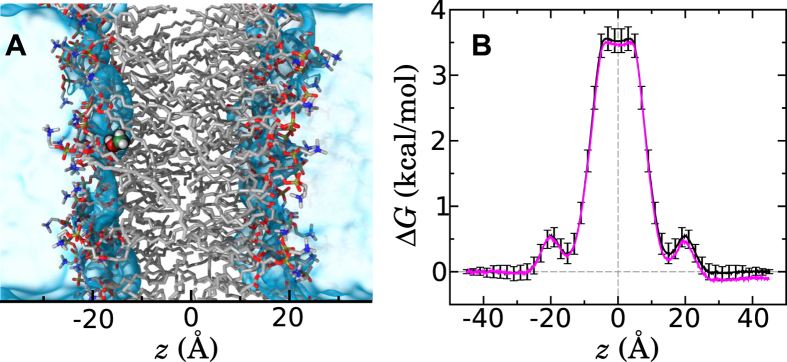
(**A**) Snapshot of a portion of the simulation system. Methanol is depicted as spheres, with H, C, and O atoms colored respectively in white, green, and red. The lipids are shown as sticks, with C, N, O, and P atoms in gray, blue, red and gold. Lipid hydrogen atoms are not shown. Although the simulation included explicit water molecules, here, for clarity, water is represented as a translucent turquoise surface. **(B)** Free-energy profile for the translocation of methanol across a fully hydrated palmitoyl-oleoyl-phosphatidylcholine bilayer. The transition coordinate, *z*, is the distance between the mid-plane of the bilayer and the center of mass of the methanol molecule. The raw free-energy profile (magenta curve) and that obtained from the antisymmetrized gradient (black curve), taking advantage of the symmetry of the pure bilayer, are compared. The error bars represent the estimated uncertainty (see Methods) of the free-energy for moving the methanol molecule from a position far from the membrane to the position *z*.

The free-energy profile of Fig. 1 appears qualitatively consistent with those previously reported for ethanol^21^,^29^, possessing small local maxima near the headgroups of the membrane (|*z*| ≈ 20 Å), minima at the headgroup–tail interface (|*z*| ≈ 14 Å), and a relatively broad barrier in the hydrophobic core of the membrane, with a small local depression near *z* = 0. Using the same lipid and force field, we calculated a barrier of 2.9 ± 0.2 kcal/mol for ethanol as compared to 3.5 ± 0.2 kcal/mol for methanol, congruent with the greater hydrophilicity of methanol. The barrier for methanol calculated here is similar to that obtained by Orsi *et al.*^32^ using a multiscale model of a dimyristoylphosphatidylcholine (DMPC) bilayer (≈3.3 kcal/mol). Bemporad *et al.*^23^ report a larger barrier (≈5.5 kcal/mol); however, their results may not be comparable since their simulations were 180 times shorter and used a different lipid and force field.

### Diffusivity assuming classical diffusion

**Figure 2:**
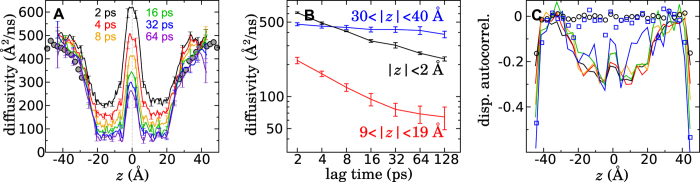
Calculated diffusivity and its dependence on the lag time. **(A)** *D*(*z*) as calculated by the Bayesian scheme described in the text for several different lag times, Δ*t*. For comparison, gray-filled symbols show *D*(*z*) as calculated from the mean squared displacement of methanol molecules in a simulation containing an aqueous solution of 5% methanol by mass. **(B)** Variation of the mean *D*(*z*) on three different regions of *z* as a function of the lag time. Note that both axes have logarithmic scales. Error bars are standard errors. **(C)** Normalized correlation of consecutive methanol displacements as a function of position *z*. In the simulations from which this was calculated, the diffusion occurred on an effectively flat free-energy landscape (obtained by applying the negative of the PMF shown in Fig. 1). The lag time between the displacements is indicated by the same color scheme used in panel A. For reference, the symbols show the correlation of consecutive displacements for an ideal Brownian dynamics trajectory of equal length with *D*(*z*) equal to that shown in panel A for a lag time of 64 ps.

It is instructive to begin by assuming classical diffusion, i.e., that the probability of methanol to move from position *z*_1_ at time *t*_1_ to position *z*_2_ at time *t*_2_. obeys Equation 2, and see whether any contradiction emerges. As in our previous work^28^,^33^, here we employ a Bayesian scheme to infer *D*(*z*) from the importance-sampling^34^ trajectories. For simplicity, we calculate the diffusivity from 13 distinct trajectories (each about 36 ns in length) in which the bias at the end of the ABF calculations was applied, but no longer updated, to yield an approximately flat, time-invariant free-energy profile along *z*. However, as shown in Figure S2 of the SI, similar results can be obtained from trajectories with nonuniform free-energy landscapes. The full scheme is described in detail in Methods. In a nutshell, the observed molecular dynamics trajectory, *Z*(*t*), is decomposed into a series of displacements over lag times Δ*t*. As shown below, this choice of Δ*t* has a substantial influence for the classical diffusion model. The probability of each displacement is calculated given an initial guess for the model parameter *D*(*z*) = 200 Å^2^/ns. In stark contrast with our past work, however, we do not use a small-displacement approximation^33^ of the Smoluchowski diffusion equation (Equation 2), but instead calculate a numerical solution to this equation using the Crank-Nicolson approach^35^, to yield the probability of arriving at the final position *Z*(*t*_*j*_ + Δ*t*) given an initial probability distribution concentrated at *Z*(*t*_*j*_), at the initial time *t*_*j*_. The equation is solved with reflecting boundary conditions (zero-flux) at ±45 Å, consistent with the confinement of the permeant to |*z*| < 45 Å during the simulation. The calculation above gives the likelihood of the observed trajectory assuming a fixed *D*(*z*), that is *P*[*Z*(*t*)|*D*(*z*)]. To quantify the degree to which different choices of *D*(*z*) are consistent with the observed trajectory, we use Bayes’ theorem and the appropriate prior for *D*(*z*) to reverse this conditional probability and obtain *P*[*D*(*z*)|*Z*(*t*)], the probability of the model parameter, *D*(*z*), given the observed trajectory. The function *D*(*z*) is then sampled by a Monte Carlo procedure^36^ to yield the posterior distribution, *P*[*D*(*z*)|*Z*(*t*)], which gives the *D*(*z*) functions most consistent with the trajectory. The mean of these functions is shown in Fig. 2A.

To validate the Bayesian scheme, we first consider a comparison to the MSD method^37^, which is simpler, but of more limited applicability. The latter method, the results of which are shown as filled symbols in Fig. 2A, is valid when neither *w*(*z*) nor *D*(*z*) vary too rapidly and can, therefore, be applied safely within the aqueous medium, i.e. |*z*| > 30 Å. We find that the Bayesian scheme and MSD method are in rough agreement for all lag times, Δ*t*. Far from the membrane, *D*(*z*) ≈ 480 Å^2^/ns, in agreement with the diffusivity determined from a simulation of a single methanol molecule in a box of water. It should be noted that this value is more than twice the value measured experimentally at a similar pressure and temperature^38^, which is not surprising since the TIP3P water model, standard to the CHARMM36 force field used in our simulations, notoriously overestimates the self-diffusion coefficient by a similar factor^39^. As the membrane is approached, the diffusivity drops abruptly, which likely stems from hydrodynamic interactions with the membrane^40^.

In order to have a consistent model of classical diffusion, the diffusivity should display no dependence on the lag time for the timescales relevant to the problem at hand^12^. However, Fig. 2A reveals that the calculated *D*(*z*) clearly depends on Δ*t* over timescales ranging from 2 to 64 ps. This dependence of the calculated diffusivity on the lag time has been identified previously^21^,^22^,^28^. The question then becomes—*which diffusivity values are the correct ones for calculating the permeability?* A convenient solution consists in calculating *D*(*z*) using the longest feasible lag time. In our case, due to sampling limitations and the ambiguity of measuring the diffusivity near the reflecting boundary, our results degrade for Δ*t* 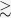  128 ps, so perhaps Δ*t* = 64 ps would be the best choice. Another option would consist in extrapolating the diffusivity to long times. However, as shown in Fig. 2B, *D*(*z*) within two different regions of the membrane appears to obey a power law over a large range of lag times. Near the center of the membrane (|*z*| < 2 Å), the average diffusivity follows the power law 〈*D*(*z*)〉 ~ Δ*t*^−0.24^ at all obtainable lag times, including 128 ps. In the appropriate limit^41^, therefore, this power law implies a mean squared displacement of 〈Δ*Z*^2^〉 ~ *t*^0.76^. Farther from the center, but still within the hydrophobic region (9 < *z* < 19 Å), an even stronger lag-time dependence is evident, suggesting a mean squared displacement of 〈Δ*Z*^2^〉 ~ *t*^0.65^. Extrapolating these power laws to very long times yields arbitrarily small *D*(*z*), which is not physically meaningful since the permeant eventually diffuses into the aqueous phase, where classical diffusion becomes valid.

### Evidence of subdiffusion

The lack of an unambiguous value of *D*(*z*) in Fig. 2B is a strong indicator of anomalous diffusion^12^,^41^. However, one might argue that the behavior exhibited here could be an artifact of the Bayesian scheme used to obtain *D*(*z*). On the contrary, Fig. 2C demonstrates that the observed subdiffusive behavior is intrinsic to the methanol trajectory. We plot for each final position, *z*, the mean correlation, *C*(*z*, Δ*t*), between the displacements over the last two Δ*t* intervals. Specifically, this correlation was computed as,


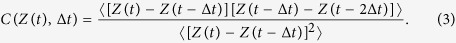


While this correlation is approximately zero in the aqueous medium (|*z*| > 30 Å), consecutive displacements of methanol within the membrane are significantly anticorrelated. This anticorrelation is evident over a wide range of Δ*t* values from 2 to 32 ps, beyond which *C*(*z*, Δ*t*) becomes too noisy to be useful. Such negative correlation is a hallmark of subdiffusion^42^. The symbols in Fig. 2C show that an ideal Brownian dynamics trajectory of equal length (using the position-dependent diffusivity shown Fig. 2A for Δ*t* = 32 ps) yields approximately zero correlation across the entire system, except near the reflecting boundaries. Thus, the negative correlation of consecutive displacements observed in the trajectory is not consistent with Markovian classical diffusion.

**Figure 3:**
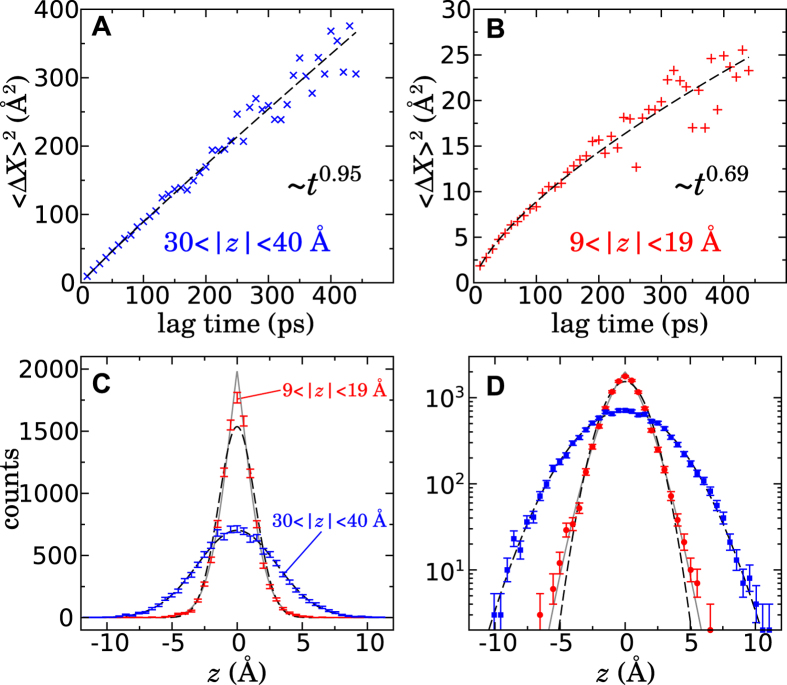
Subdiffusion of methanol within the membrane. Mean squared displacement of methanol parallel to the membrane (along the *x* axis) within the aqueous phase **(A)** and within the membrane **(B)**. The dashed black curves are power-law fits to the data. Histogram of methanol displacements along the *x* axis for a lag time of 10 ps on a linear (**C**) or logarithmic (**D**) scale. Dashed black lines are Gaussian curves with the same mean and variance as the histograms derived from the simulations, while the gray curve is a fit of the subdiffusive probability distribution given by Metzler and Klafter^10^. The error bars are calculated as described by Heinrich^43^, detailed in the caption of Figure S9 of the SI.

A standard measure of subdiffusive behavior consists in analyzing the MSD as a function of lag time^10^, where a linear *t*-dependence implies classical diffusion, while a power law *t*^*α*^ for *α* < 1 implies subdiffusion. For bilayer permeation, this prescription is complicated by the fact that the diffusivity depends on *z*, making the MSD nonlinear, even in the case of classical diffusion on a flat PMF. An alternative is to consider displacements parallel to the membrane, for example, along the *x* axis, while maintaining a fixed interval along *z*. This alternative assumes the diffusivity is isotropic and that the displacements in *x* and *z* are independent. Within the membrane, we do find a small positive correlation (*r* ~ 0.1) between simultaneous displacements along *x* and *z*, which we shall ignore as its effect appears to be small. For comparison, displacements along *z* have also been analyzed (Figure S9 of the SI) for short lag times (10 ps) and exhibit similar behavior to that shown in Fig. 3 for lateral displacements. Therefore, in Fig. 3, we consider trajectories *X*(*t*) along the *x* axis, in which the *z*-position is confined to two different regions where *D*(*z*) shows little variation—namely, in the aqueous phase, both far from the membrane and the reflecting boundary (30 < |*z*| < 40 Å), and a region in the membrane 9 < |*z*| < 19 Å. Figure 3A shows that 〈Δ*X*^2^〉 is approximately linear in the aqueous phase on times ranging from 10 to 450 ps. A power-law fit of 〈Δ*X*^2^〉 gives an exponent of 0.95, essentially consistent with classical diffusion in the aqueous phase. On the other hand, 〈Δ*X*^2^〉 is clearly nonlinear within the membrane (9 < |*z*| < 19 Å), where we find a trend of 〈Δ*X*^2^〉 ~ 0.69, well within the subdiffusive regime.

Another hallmark of subdiffusion is a non-Gaussian form of the spatial probability distribution of the diffusing particle. As shown in Fig. 3C, this distribution in the aqueous phase is very nearly Gaussian, while that for 9 < |*z*| < 19 Å appears to exhibit the cusp-like peak typical of solutions of fractional diffusion equations in the subdiffusive regime. Metzler and Klafter^10^ give an infinite series (Eq. 46) and asymptotic expression (Eq. 45) for the probability distribution, *W*_*α*_(*x*, *t*), of a diffusing particle on a flat energy landscape in the subdiffusive regime. A fit of this distribution to the simulation data was calculated by maximizing


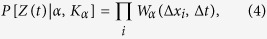


where Δ*x*_*i*_ were the observed displacements of methanol. The fits for 9 < |*z*| < 19 Å and 30 < |*z*| < 40 Å gave *α*-values of 0.74 and 0.98. The cusp of the former fit is somewhat sharper than that in the observed histogram, but this behavior might be ascribed to fuzziness of the definition of center of membrane^30^. The logarithmic plots in Fig. 3D suggest a long-tailed distribution for 9 < |*z*| < 19 Å, a further indication of subdiffusion^12^. The fit of the subdiffusive model is statistically consistent with the observed histogram for 9 < |*z*| < 19 Å, while the Gaussian fit lies outside the error bars^43^ for this same interval. To quantify the degree to which the histograms are non-Gaussian, we performed the Shapiro-Wilk test of normality^44^, as implemented in the software R^45^, for which a *p*-value > 0.5 indicates that a Gaussian distribution is likely. For 9 < |*z*| < 19 Å, we obtain *p*-values ranging from 10^−16^ to 10^−6^ over lag times spanning 10 to 130 ps, a strong indication of non-Gaussian behavior. In stark contrast, for 30 < |*z*| < 40 Å, the corresponding *p*-values range from 0.3 to 0.9.

An additional strategy to characterize diffusion within the membrane might be to construct a homogeneous system (without the complications arising from the intrinsic anisotropy of the bilayer) that possesses some properties of the membrane core. We have pursued this route by performing simulations of methanol diffusion in a periodic box of liquid hexadecane, as detailed in Figure S10. Although there is no rigorous link between diffusion of methanol in this system and that in the membrane, this strategy furnishes some useful insights. Namely, it exhibits MSD distributions with clear cusps and long tails that best fit theoretical MSD distributions^10^ with *α* in the range of 0.7 to 0.8 for lag times between 2 and 64 ps.

### Physical origin of subdiffusion

**Figure 4:**
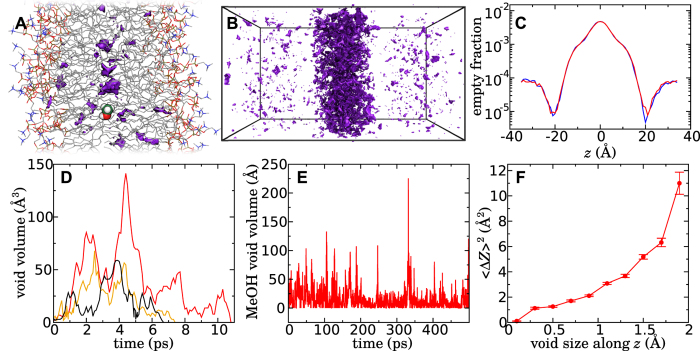
Voids within the membrane and their effect on methanol motion. **(A)** Snapshot of a molecular dynamics simulation of methanol permeation with spontaneously appearing void regions highlighted in violet. **(B)** Overlay of the voids in 30 randomly selected snapshots extracted from simulations. **(C)** Fraction of empty volume as a function of *z*. The blue and red curves, respectively, were calculated from trajectories in the absence of methanol and with methanol in the interval −5 < *z* < 5 Å. **(D)** Evolution of three exemplary large voids in the membrane. The volume of each void is plotted as a function of time from its first appearance. **(E)** Volume of the void immediately surrounding the methanol molecule during a simulation in which the alcohol occupied the membrane. If there is no empty space adjacent to methanol, then the volume was recorded as zero. Methanol atoms were ignored in computing this volume. **(F)** Mean squared displacement of methanol along the permeation axis as a function the void size along this axis. The plot represents displacements with a lag time of Δ*t* = 30 ps, averaged over four 1 ns simulations in which the alcohol diffused within the membrane and a bias was applied to yield a flat free-energy profile along *z*. This void size was calculated as the standard deviation of the positions of the voxels forming the void. The error bars represent standard errors.

In the previous section, we have provided ample evidence that motion of methanol through the membrane is subdiffusive, while exhibiting classical diffusion in the aqueous medium. *What is the physical reason for this difference in behavior?* A high fraction of empty space near the center of the bilayer relative to that in bulk water has been recognized in molecular simulations for more than two decades^6^,^46^. We find that many discrete, low-density regions, or voids, contribute to this empty space, which are identified algorithmically as described in Methods. These voids, examples of which are shown in Fig. 4A,B, form spontaneously both in the presence and absence of the permeant, and rapidly fluctuate in and out of existence. It ought to be noted that these voids are truly empty, containing neither lipid moieties nor water molecules. Although such voids appear in the aqueous medium, they tend to be much smaller and much more infrequent, as can be seen in Fig. 4B,C. It is also important to note that the voids occupy only a small amount of space. Even at the center of the membrane, where the density of voids is the greatest, voids constitute less than 1% of volume, as is evident in Fig. 4C. It should be noted that simulations of bulk liquid hexadecane exhibit voids of similar prevalence and size (see Figure S10A of the SI).

Within the membrane, large voids, reaching volumes of about 200 Å^3^ and extending as much as 10 Å along the *z* axis appear infrequently, but seem to have a large effect on the permeation of methanol. A movie showing void-mediated diffusion of methanol through the membrane is included in the SI. Figure 4D shows the evolution of the three exemplary large voids, which persist for several picoseconds, fluctuating in size, before disappearing completely. The permeant encounters many voids as it diffuses in the membrane. Figure 4E shows an example trajectory of the empty volume surrounding the methanol molecule. Encounters with particularly large voids (volume >100 Å^3^) are rare events, which happen only a few times per nanosecond. When not in contact with a void, or in contact with a very small void, the methanol displacements are very small, as revealed by Fig. 4F. The MSD rises rapidly as the extent of the voids along the the *z* axis is increased. Further evidence of this dependence of the displacement on the void size is shown in Figure S3 of the SI, where we see that the mean squared displacement increases with decreasing atomic density in the neighborhood of the permeant. We, therefore, conclude that displacements of methanol are small in the absence of voids and that the most significant displacements occur due to rare, large voids that spontaneously appear near the permeant. Thus, Fig. 4E can be seen as a source of correlated noise that drives methanol diffusion and is punctuated by rare events, thereby providing a plausible explanation for the emergence of subdiffusion. It should be noted that the motion of the permeant also depends somewhat on the presence of coordinating water molecules. Figure S4 of the SI reveals that methanol is usually accompanied by one or more coordinating water molecules for |*z*| > 6 Å, which are linked with a reduction in the magnitude of typical displacements.

**Figure 5:**
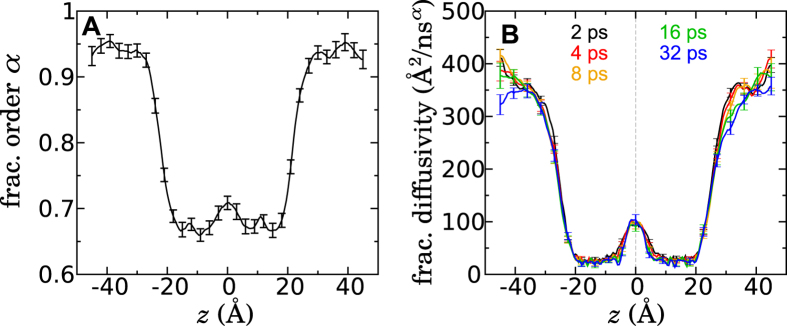
Estimated parameters of the fractional Smoluchowski model. **(A)** Fractional order *α* as a function of position, as calculated by the Bayesian scheme. **(B)** Fractional diffusivity, *K*_*α*_(*z*), as calculated by the Bayesian scheme for several lag times, Δ*t*.

It is likely that the formation of voids is linked with lateral diffusion of lipids. The lateral motion of lipids on the timescales relevant for methanol permeation is subdiffusive^18^; thus, the permeant motion might acquire a similar character. It may therefore not be coincidental that the fractional order determined here in the core of the membrane ( ≈ 0.7, see Fig. 5) is similar to that calculated^18^ for the lateral membrane diffusion of a lipid molecule’s center of mass.

We recognize, therefore, two regimes of methanol diffusion: one in which the permeant rattles within a relatively fixed configuration of lipids and another involving larger time and length scales controlled by major rearrangements of the membrane lipids and associated with void formation within the membrane. As has been noted^47^, the transition from a fast diffusion regime at short time and length scales to a slower regime at longer time and length scales requires an intermediate subdiffusive regime. We hypothesize that subdiffusion in the membrane permeation may result from just such a transition. It has been argued that such subdiffusion is only “apparent”^47^; however, our goal is to construct a consistent model, and, as we show below, models of anomalous subdiffusion appear to describe well the motion of methanol on timescales approaching the typical permeation time.

### The fractional Smoluchowski model

Here, we propose to model the motion of methanol in the membrane using a time-fractional^48^ Smoluchowski diffusion equation, similar to Equation 2, except that the first-order time derivative has been replaced by a Caputo^11^ derivative of fractional order *α*(*z*),


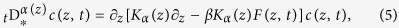


where the fractional order, *α*(*z*), is position-dependent, consistent with our findings showing classical diffusion in the aqueous solution outside of the membrane, and varying degrees of subdiffusion within. The position-dependent diffusivity of classical diffusion, having dimensions of *L*^2^/*T*, is replaced with *K*_*α*_(*z*), with units *L*^2^/*T*^*α*^. To estimate this fractional diffusivity, we apply the Bayesian scheme described above to the molecular dynamics trajectory, numerically solving Equation 5 with the method of Sweilam *et al.*^48^ to compute the probability of the observed displacements. First, we allowed both the fractional order, *α*(*z*), and *K*_*α*_(*z*) to be optimized by the Bayesian scheme. To allow *α*(*z*) and *K*_*α*_(*z*) to be determined unambiguously, we first optimized both these functions using trajectory data with Δ*t* = 4 and 8 ps. Figure 5 shows the resulting *α*(*z*). The Bayesian scheme was then repeated (assuming a symmetrized and smoothed version of this *α*(*z*)) to obtain *K*_*α*_(*z*) for different lag times, which are depicted in Fig. 5.

As expected from the results above, *α*(*z*) approaches one (or at least 0.95) at large distances from the membrane, indicating nearly classical diffusion of methanol in water, while taking on smaller values in the membrane, unambiguously in the subdiffusive regime. *α*(*z*) reaches its smallest values (≈0.67) for 4 < |*z*| < 15 Å, consistent with the region of strongest negative correlation in Fig. 2C. A value of *α* ≈ 0.7 near the core of the membrane roughly agrees with the results presented in Figs 2B,C and 3B.

With the fractional model, we observe much less variation of *K*_*α*_(*z*) with the lag time than we did for *D*(*z*) in both the absolute and relative senses. Hence, the fractional Smoluchowski model appears to offer a much more consistent description of permeant motion than the conventional Smoluchowski model. The fractional model can, therefore, be constructed using a wide range of lag times, so that the most convenient one can be chosen. It is often more convenient to utilize small values for the lag time because one can generate many short trajectories in parallel, as opposed to a few long ones. Furthermore, ambiguity in the results due to the presence of reflecting boundaries are less severe for shorter lag times, making it easier to use trajectories from typical stratified free-energy calculations.

### Permeability calculation

For the classical diffusion model, the permeability can be efficiently computed from Equation 1. Using the diffusivity calculated for Δ*t* = 64 ps (see Fig. 2), we obtain a permeability of 0.317 cm/s for this model. However, Equation 1 is not valid for the fractional model, which can be clearly seen from the fact that fractional diffusivity *K*_*α*_(*z*) possesses different units than *D*(*z*). As detailed in Methods, another approach to obtain the permeability is to solve the Smoluchowski equation with a concentration imbalance enforced between the two sides of the membrane. This approach yields the same result as Equation 1 for the classical diffusion model. Applying this approach to the fractional model, we obtain a permeability of 0.158 cm/s, approximately half that of the classical model. Given the more than twofold overestimation of the diffusivity of methanol in water by the CHARMM36 force field, one could argue that the corrected permeability should be proportionally lower; however, it is unclear whether the overestimation of methanol diffusivity in water implies an overestimation of the kinetics in the membrane as well. The permeability value computed here is similar to that previously inferred from the multiscale simulations of methanol permeation by Orsi *et al.*^32^ We saw in the “Free-energy profile” subsection that the atomistic simulations of Bemporad *et al.*^23^—using much less simulated time, a temperature of 323 K, and an older CHARMM force field—gave a larger free-energy barrier; thus, it is not surprising that they give a permeability an order of magnitude smaller than that calculated here. Ly and Longo^49^, using 1-stearoyl, 2-oleoyl phosphatidylcholine (SOPC), bilayers have quoted a permeability four orders of magnitude smaller. However, the interpretation of these experiments has been questioned^21^, and the value is several orders of magnitude smaller than the experimental permeability for water through pure lipid bilayers^50^, which appears inconsistent with the chemical natures of the two molecules. The results of Brahm^51^ are closer to the computationally predicted values, but cannot be directly compared since they involved the multicomponent membranes of human red blood cells.

### Validation: Unbiased diffusion from the origin

**Figure 6:**
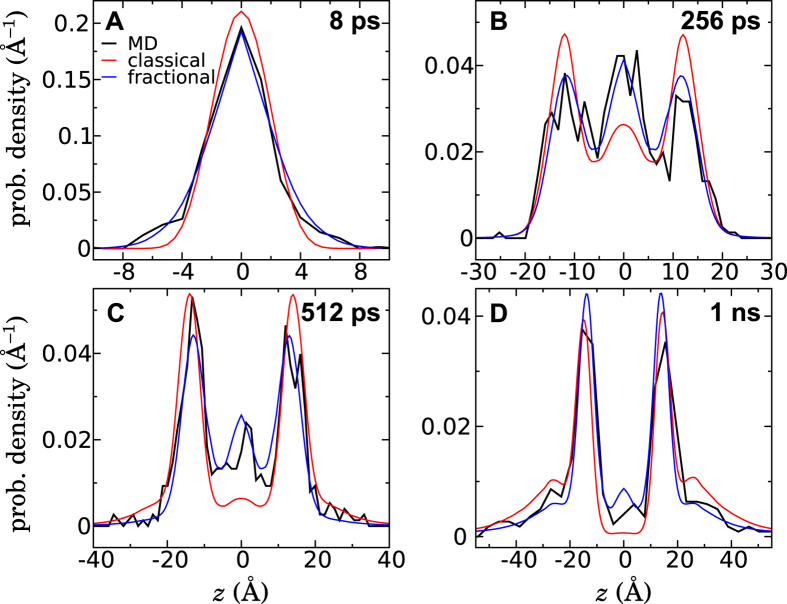
Unbiased diffusion of methanol from the center of the membrane. **(A–D**) Position distribution of methanol at various times derived from simulations and from the classical and fractional Smoluchowski models. The curves show histograms over 570 independent molecular dynamics simulations (black), predictions of the classical Smoluchowski model based on *w*(*z*) as computed by the ABF method and *D*(*z*) calculated by the Bayesian scheme with a lag time of 64 ps (red), and predictions of the fractional Smoluchowski model based on *w*(*z*) as computed by the ABF method and *α*(*z*) and *K*_*α*_(*z*) calculated by the Bayesian scheme (blue). For the fractional model, the choice of the lag time at which *α*(*z*) and *K*_*α*_(*z*) were inferred makes little difference, at least for the range of times considered in this work.

The fractional Smoluchowski model shown in Fig. 5 was constructed from a simulation where the underlying free-energy landscape was canceled out to obtain adequate sampling and simplify the analysis. One might, therefore, wonder whether this model will remain valid in the real, unbiased system. To answer this question, we performed 570 independent simulations to track the evolution of the probability distribution of a single methanol initially positioned near the origin, with no bias applied. Each of the simulations began with a distinct atomic configuration (in which |*Z*(*t* = 0)| < 0.3 Å) and random initial velocities. Figure 6 shows the methanol probability distributions derived from these simulations at four different times. The qualitative evolution of this distribution can be understood by considering the PMF in Fig. 1. The alcohol first essentially undergoes free diffusion where the PMF is relatively flat (|*z*| < 3 Å). Beyond this point, it is driven out of the membrane by a mean thermodynamic force, until reaching the metastable minima at *z* ≈ ±14 Å. Owing to its amphipathic nature, the alcohol tends to occupy these minima for some time before being expelled into the aqueous solution.

For reference, Fig. 6 shows the results of solving the classical Smoluchowski diffusion equation on symmetrized versions of *w*(*z*) (Fig. 1) and *D*(*z*) for Δ*t* = 64 ps (Fig. 2), the longest lag time for which we obtained reliable results. The methanol distribution derived from the fractional model is shown in Fig. 6, calculated using symmetrized versions of the *α*(*z*) and *K*_*α*_(*z*) functions depicted in Fig. 5. The classical diffusive model fails to reproduce the results of the simulations in many respects, which is not surprising given the evidence of anomalous diffusion that we have already pointed out. In contrast, the fractional Smoluchowski model appears to provide a statistically accurate picture of methanol diffusion on timescales ranging from 1 ps to 1 ns. For instance, the distribution observed at *t* = 8 ps (Fig. 6A) agrees well with the fractional Smoluchowski solution, but has heavier tails and a narrower peak than the classical solution. Anomalously long residence times at the initial position, a characteristic of subdiffusion^10^, are evident in both the molecular dynamics results and the fractional model. The classical model, on the other hand, significantly underestimates the probability of staying near the initial position for *t* = 256 ps and 512 ps (Fig. 6B,C).

The timescales on which we have identified subdiffusive motion, 1 ps to 1 ns, are relevant for the permeation process, since methanol can move between different regions of the membrane on these times. Further evidence of the importance of this timescale are provided by the simulations of Patra *et al.*^52^, which reveal that ethanol permeation events take place in about 300 ps. Given that we calculate the free-energy barrier for methanol to be about 0.6 kcal/mol higher than that for ethanol, we can expect methanol permeation to take place on the ~1 ns timescale.

The fractional model assumes a time-invariant fractional order *α*. However, simulations of methanol diffusion in hexadecane, detailed in Figure S10 of the SI, show a subdiffusive regime on the timescale range of 1 ≤ Δ*t* ≤ 256 ps, approaching a slow classical diffusion regime at longer times. In the membrane, the subdiffusive regime appears to persist at longer times (at least approaching 1 ns), which is not unexpected since liquid hexadecane is not an exact model of the membrane core. On the other hand, it is conceivable that the value of *α* within the membrane varies with the lag time and may tend toward the classical limit (*α* = 1) for sufficiently long times. Such a long-timescale classical regime, analogous to that observed for methanol diffusion in hexadecane, can never be fully manifested owing to the finite thickness of the membrane, which ensures that most permeants exit the membrane before classical diffusion within the membrane can emerge.

## Conclusions

Here, for the first time, we have provided a model for the membrane permeation of a small molecule that does not assume a lack of long-range correlations in time and space. This model allows us to better understand permeation dynamics for molecules such as methanol, which exhibit subdiffusive behavior on the characteristic timescales of their permeation. Although the model parameters (*α*(*z*) and *K*_*α*_(*z*)) are computed from simulations in which a biasing force is applied, Fig. 6 demonstrates that the resulting model can accurately describe permeant (sub)diffusion in nature, i.e., in the absence of artificial biasing forces. However, all choices of biasing forces may not yield a consistent diffusive model, as large biases may result in hydrodynamic drag inconsistent with Equation 5. Our simulations suggest that this subdiffusive behavior is a result of permeation being governed by the spontaneous formation of voids within the membrane, which leads to intermittent large displacements of a permeant that is otherwise nearly immobile. It seems likely that the phenomena giving rise to subdiffusion in the case of methanol would also be relevant to other permeants, including therapeutic agents. A hint of this conjecture can be seen in Figure S8 of the SI, where the drug codeine is shown to exhibit trends similar to those of methanol in the lag time dependence of the classical diffusivity and the correlation of consecutive displacements. However, the importance of the subdiffusive behavior goes beyond the scope of the present work and deserves an investigation in its own right.

As detailed in Figure S10 of SI, simulations of methanol in bulk hexadecane suggest that the subdiffusive regime may emerge as a crossover^47^ between fast confined diffusion of the permeant and slower diffusion governed by lipid rearrangements and associated void dynamics. One might predict, therefore, that larger permeants, by virtue of having slower dynamics than methanol, reach the long-timescale classical diffusion regime earlier in the permeation process and are thus more easily described by a classical model. On the other hand, conditions that retard lipid dynamics, for instance, the presence of cholesterol in the membrane^53^, might extend the subdiffusive regime to longer timescales. Such predictions of permeant dynamics would be facilitated by a better understanding of the mechanisms underlying the subdiffusive behavior.

## Methods

### Molecular dynamics methods

The molecular assembly consisted of a methanol molecule placed near a lipid bilayer formed by 100 POPC molecules in equilibrium with 9,282 water molecules, corresponding to a cell dimension of about 59 × 59 × 119 Å^3^. Owing to the periodic boundary conditions, the membrane spanned the *xy*–plane in a continuous manner. All the molecular dynamics simulations reported here were performed using the parallel, scalable program NAMD 2.10^54^, using the CHARMM36 force field for lipids^55^, the CHARMM General Force Field (CGenFF)^56^ for methanol, and the TIP3P water model^57^. A momentum-conserving Lowe-Andersen thermostat^58^ was utilized to maintain the temperature at 308 K with a rate of collision of 50 ps^−1^. The pressure was maintained at 1 atm applying the Langevin piston method independently to the *z*-axis and *xy*–plane^59^. Covalent bonds involving hydrogen atoms were constrained to their equilibrium length by means of the RATTLE^60^ algorithm, except for water molecules, for which the SETTLE algorithm^61^ was applied. Long-range electrostatic forces were evaluated using the particle-mesh Ewald scheme (grid spacing 1.2 Å), while a smoothed 8–9 Å spherical cutoff 28 was used to truncate short-range van der Waals and electrostatic interactions. The r-RESPA multiple time-step algorithm was applied to integrate the equations of motion with a time step of 2 and 4 fs for short- and long-range interactions^62^.

### Free-energy calculations

The one-dimensional free-energy profile, or PMF, *w*(*z*), for methanol permeation was calculated using the ABF method^63^,^64^, as implemented in the Colvars module^65^. The transition coordinate chosen to investigate permeation was defined as the projection onto the *z*-direction of Cartesian space, i.e., the normal to the membrane, of the vector connecting the center of mass of the phosphorus atoms of the membrane to that of methanol. The permeation pathway was discretized in bins 0.1 Å wide, wherein samples of the local force acting along *z* were accrued. In addition, to improve the efficiency of the free-energy calculation^64^, the 90 Å long permeation pathway was broken down into nine strata, or windows, overlapping sequentially over 5 Å. ABF allows the use of relatively large windows (15 Å in length in the present work); this may be important because, for example, in the inner windows, −15 < *z* < 0 Å and 0 < *z* < 15 Å, the methanol molecule is able to travel from the center of the membrane to regions where water penetration is high^28^, permitting water molecules coordinating the alcohol to be exchanged (see Figure S4 of the SI). While overlap between neighboring windows does not constitute a prerequisite of the algorithm, by virtue of the continuity of the free-energy gradient across *z*, this strategy has proven necessary to ensure accurate estimates over the entire domain of the other ingredient of Equation 1, namely the position-dependent diffusivity of the substrate, *D*(*z*), as described below. Initial conditions for each window were obtained from steered molecular dynamics simulations^66^, where the alcohol molecule was slowly pulled through the membrane prior to suitable equilibration. Flat-bottom harmonic potentials were applied to confine the methanol molecule within the current window. The ABF method returns the number of force samples and the estimated gradient of the PMF for each window *s*, denoted respectively *n*_*s*_(*z*) and *g*_*s*_(*z*). The results from the windows were combined by the weighted sum 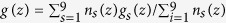, where *n*_*s*_(*z*) = 0 when *z* falls outside the domain of window *s*. The PMF was then computed by evaluating numerically the integral 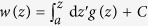. As is necessary for the application of Equation 1, we conventionally anchored *w*(*z*) at zero in bulk water; thus, the value of the constant *C* was chosen to give *w*(*z*) a mean value of zero on the interval −45 ≤ *z* ≤ −43 Å. The result is shown as the magenta line in Fig. 1. The symmetrized PMF was obtained by antisymmetrizing the gradients prior to integration by *g*_sym_(*z*) = [*n*(*z*)*g*(*z*) − *n*(−*z*)*g*(−*z*)]/[*n*(*z*) + *n*(−*z*)]. The unsymmetrized profile gives a lower bound on the statistical uncertainty in the calculation, since *w*(*z* = ±45 Å) are in principle exactly equal. The error bars in Fig. 1 were computed differently—by comparing the estimated mean forces from the first and second halves of the simulations and propagating the inferred uncertainty in these forces to the free-energy profile. Further detail is given in the “Uncertainty in free energy” section of the SI.

### Establishing the baseline diffusivity

Two additional molecular dynamics simulations were performed to validate the diffusivity calculations described below. In the first simulation, a single methanol molecule was placed in a periodic box of 4,000 water molecules (approximately a cube of (49.5 Å)^3^) and simulated for 100 ns at 308 K and 1 atm. Prior to the analysis, the center of mass of the overall system was fixed to the origin using a trajectory in which no wrapping with respect to the periodic boundaries was performed. The diffusivity of methanol in water was estimated by calculating the fit line for 〈[*Z*(Δ*t*) − *Z*(0)]^2^〉/2 versus Δ*t*, with Δ*t* ranging from 10 to 100 ps, much larger than the decorrelation time (see Figure S5 of the SI). A Pearson correlation coefficient of *r* = 0.99 demonstrates the linearity of the fit line and appropriateness of the classical diffusivity model for methanol in water on this timescale. This procedure was repeated for the *x* and *y* axes, giving a mean result of 479 ± 24 Å^2^/ns, where the uncertainty is the maximum deviation of the three values from the mean.

One should note that molecular dynamics simulations typically exhibit artifacts in diffusivity due to hydrodynamic interactions between periodic images^67^. For the bilayer system, the estimated correction is roughly −7 Å^2^/ns, which we ignored since, in most cases, it is smaller than the uncertainty of the calculations. However, the finite-size correction^67^ was somewhat more significant for the water cube system (−18 Å^2^/ns) than for the membrane system. Thus, the figure 468 ± 24 Å^2^/ns is best for comparing to the other diffusivities of this work.

The second system was similar to the POPC membrane used in the free-energy calculations, except that the *x*- and *y*-dimensions were doubled, producing a bilayer of 400 POPC molecules, and the *z* dimension was increased to ≈187 Å. Instead of pure water, the solution contained 5% by mass of methanol, totaling 2,000 alcohol molecules and 67,564 water molecules. The use of a methanol solution improved statistics while not substantially altering the kinetic properties of the solution. The system was equilibrated for 2 ns and run for an additional 4 ns for calculation of the diffusivity. We calculated the diffusivity of the methanol molecules in water far from the membrane (|*z*| > 55 Å) by *D* = 〈[*Z*(Δ*t*) − *Z*(0)]^2^〉/(2Δ*t*) for Δ*t* = 30 ps, obtaining 481 ± 4 Å^2^/ns, consistent with the result for the water box system.

### Bayesian inference of the classical diffusivity

For simplicity of interpretation, we calculated the diffusivity using trajectories from simulations where the effective free-energy landscape was rendered flat (*w*_eff_(*z*) = 0), canceling the PMF calculated above by applying the force +∂_*z*_*w*(*z*) to the center of mass of methanol. Moreover, no time-dependent bias was applied. The molecular model was the same as that used for the free-energy calculations, namely 100 lipids and 9,282 water molecules. In these additional simulations, the alcohol molecule was confined within the full interval −45 ≤ *z* ≤ 45 Å, using a flat-bottom potential. Thus, in what follows, we will consider Equation 2 with *F*(*z*, *t*) effectively equal to zero.

As in our previous work^28^,^33^, we first calculate the likelihood *P*[*Z*(*t*)|*D*(*z*)], i.e. the probability of the observed trajectory, *Z*(*t*), given an initial guess for the model parameter *D*(*z*), which is represented by a cubic interpolant with continuous first derivatives (grid spacing *h* = 0.5 Å). The trajectory is split into many displacements over time periods Δ*t*, so that this likelihood is calculated as a product over the probability for each displacement from *Z*(*t*_*j*_) to *Z*(*t*_*j*_ + Δ*t*),


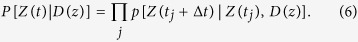


To avoid overflow in the machine representation, our code actually calculates the cost, −ln(*P*[*Z*(*t*)|*D*(*z*)]), transforming the above product into a sum.

A novel aspect of the present work is that we numerically solve Equation 2, using the Crank-Nicolson approach^35^ to obtain an accurate estimate of the displacement probability on timescales over which *D*(*Z*(*t*)) and *w*(*Z*(*t*)) vary considerably. As can be seen in Figure S6 of the SI, the approximate Gaussian form of the probability distribution that we used in our previous work^28^,^33^, depending on the values of *D*(*z*) and ∂_*z*_*D*(*z*) only at the initial point *Z*(*t*_*j*_), gives similar results, compared to the Smoluchowski solution up to Δ*t* = 4 ps. For larger lag times, Δ*t*, significant discrepancies become visible in regions of large *D*(*z*), namely in the aqueous phase and near *z* = 0.

The Smoluchowski diffusion equation was solved using the Crank-Nicolson prescription on the domain −45 ≤ *z* ≤ 45 Å, with a grid spacing of *h* = 0.5 Å, the initial condition *c*(*z*_*i*_, *t*_*j*_) = 1/*h*, equivalent to all probability being concentrated at the node, *i*, nearest to the initial position *Z*(*t*_*j*_). This way, the solution had to be calculated only once for all segments of the trajectory with the same initial node for a given *D*(*z*). Robin (zero-flux) boundary conditions, i.e. *D*(*a*)∂_*z*_*c*(*a*, *t*) − *βD*(*a*)*F*(*a*, *t*)*c*(*a*, *t*) = 0, were implemented at the boundaries *a* = ±45 Å. Equation 2 was evolved in time by Δ*t*/*τ* steps to obtain the final probability distribution. The probability of the displacement from *Z*(*t*_*j*_) to *Z*(*t*_*j*_ + Δ*t*) over the time Δ*t* was thus taken to be the amplitude of the probability density at the node, *f*, nearest to the final point, i.e. *p*[*Z*(*t*_*j*_ + Δ*t*)|*Z*(*t*_*j*_), *D*(*z*)] ∝ *c*(*z*_*f*_, *t*_*j*_ + Δ*t*). The tridiagonal matrix representing the Crank-Nicolson system was solved using the GNU Scientific Library^68^. All calculations shown here used a grid spacing of *h* = 0.5 Å and an integration time of *τ* = 500 fs. To validate this choice, we also tested *h* = 0.2 Å and *τ* = 100 fs, which was much more computationally costly, yet yielded essentially identical results (see Figure S7 of the SI).

With the likelihood *P*[*Z*(*t*)|*D*(*z*)] calculated via Equation 6, the desired posterior probability was obtained by,


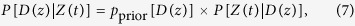


where the prior reflected an assumption of scale invariance and smoothness of the diffusivity^9^, being a product of


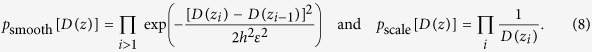


Here, the smoothness was only weakly restrained with *ε* = 50 Å/ns. The posterior distribution *P*[*D*(*z*)|*z*(*t*)] was sampled by the Metropolis-Hastings algorithm^36^. The initial guess for *D*(*z*) was uniform, *D*(*z*) = 200 Å^2^/ns. A modification of *D*(*z*) was attempted at each iteration of the algorithm by randomly choosing a node *D*(*z*_*i*_) and shifting its value by *sT*_*k*_, where *s* = 2 Å^2^/ns and *T*_*k*_ was selected from the long-tailed distribution 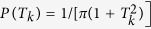. For each calculation, 10000*n* modifications were attempted, where *n* = 181 was the number of nodes for *h* = 0.5 Å. The acceptance rate of the Monte Carlo modifications varied between 0.6 and 0.8. In all cases, the posterior probability converged after about 2000*n* steps, fluctuating thereafter about a mean value. The configuration of *D*(*z*) was stored every 10 steps. The *D*(*z*) values shown in Fig. 2 were calculated by averaging over the last 4/5 of the stored samples, discarding those from the convergence phase. The C++ code for performing these calculations has been made publicly available at http://github.com/jeffcomer/DiffusionFusion.

### Identification of voids

Empty regions within the system were identified by mapping atomic configurations from the molecular dynamics trajectories onto a grid. The entire periodic cell was filled with a three-dimensional grid with a uniform spacing of approximately 0.5 Å. Periodic boundary conditions were enforced in the marking of empty regions. Each non-hydrogen atom was considered to be a sphere of radius equal to *R*_min_/2 as defined in the CHARMM36 force field^56^, plus an additional probe radius 1.5 Å. Inclusion of hydrogen atoms was also examined; it gave similar results (i.e. a much larger density of voids in the membrane than in the aqueous phase), while being more computationally costly. All voxels of the grid were marked as being occupied by an atom or empty, using a cell decomposition for efficiency. The empty grid voxels were then subjected to a flood-fill algorithm to identify contiguous voids. For the purpose of the flood fill, two grid voxels were considered to be connected if they shared any vertex, i.e. each voxel had 26 neighbors. The size of each distinct void along the *z* axis was characterized by the standard deviation of the void distribution along *z*:


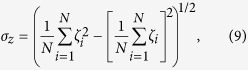


where the sums run over all voxels *i* in a contiguous empty region, and *ζ*_*i*_ = *I*(*z*_*i*_ − *z*_1_) + *z*_1_ and the function *I*(Δ*z*) gives the minimum distance between two *z*-values consistent with the periodic boundary conditions.

### Bayesian inference of the fractional diffusivity

A major advantage of the Bayesian inference scheme lies in its flexibility to accommodate different diffusive models^9^,^29^,^33^,^69^. Thus, the Bayesian scheme for the fractional diffusion model was nearly identical to that employed for the Markovian model, except that *p*[*Z*(*t*_*j*_ + Δ*t*)|*Z*(*t*_*j*_), *D*(*z*)] was calculated based on the time-fractional Smoluchowski equation, Equation 5. The solution was obtained using the Crank-Nicolson approach o time-fractional diffusion equations detailed by Sweilam *et al.*^48^. Determining the fractional Smoluchowski solution is considerably more computationally expensive than the conventional solution on account of the long memory of the Caputo fractional derivative^11^, which, at each time step, involves a sum over the solutions obtained at all previous time steps (Equation 5 of Sweilam *et al.*)^48^. More efficient approximations may exist, but were not considered in the present work.

### Calculation of the permeability

In Equation 2, the particle current can be identified as *J*(*z*, *t*) = −[*D*(*z*)∂_*z*_ − *βD*(*z*)*F*(*z*, *t*)]*c*(*z*, *t*), where the first term is is referred to as the diffusion current and the second term is the drift current. In the steady state, the current through the system, *J*_steady_, is a constant, independent of *z* and *t*. Therefore, another route to obtain the permeability, distinct from that embodied in Equation 1, consists in numerically evolving Equation 2 or 5 with a small imbalance in concentrations, Δ*c*, enforced on each side of the membrane, until *J*(*z*, *t*) converges, i.e., the steady state is attained. This enforcement of a fixed concentration at the boundaries can be formulated as the Dirichlet boundary conditions, *c*(−*L*/2, *t*) = *c*_0_ + Δ*c* and *c*(*L*/2, *t*) = *c*_0_, where *L* is the length of the system and *c*_0_ ≫ Δ*c* is an arbitrary concentration. The small concentration imbalance drives a small net current through the system in the steady state, which, similar to Ohm’s law in electricity, yields a resistance to permeation given by *R* = Δ*c*/*J*_steady_ and, thus, the permeability by *P* = *J*_steady_/Δ*c*. For the fractional model, *J*(*z*, *t*) can be straightforwardly calculated in the aqueous portion of the system where *α*(*z*) ≈ 1.

## Additional Information

**How to cite this article**: Chipot, C. and Comer, J. Subdiffusion in Membrane Permeation of Small Molecules. *Sci. Rep.* **6**, 35913; doi: 10.1038/srep35913 (2016).

**Publisher’s note**: Springer Nature remains neutral with regard to jurisdictional claims in published maps and institutional affiliations.

## Supplementary Material

Supplementary Video

Supplementary Information
