# Supplementary material for: Subdiffusion in Membrane Permeation of Small Molecules
Source: Sci Rep. 2016 Nov 2;6:35913. doi: 10.1038/srep35913 (PMC5090971; doi:10.1038/srep35913)
Supplement: Supplementary Information [file srep35913-s2.pdf]

Supplementary Information for Subdiffusion in Membrane Permeation of Small Molecules

Christophe Chipot[1,2,3] and Jeffrey Comer[4,*]

[1]Laboratoire International Associé Centre National de la Recherche Scientifique et University of Illinois at Urbana-Champaign, Unité Mixte de Recherche n°7565, Université de Lorraine, B.P. 70239, 54506 Vandœuvre-lès-Nancy cedex, France
[2]Theoretical and Computational Biophysics Group, Beckman Institute for Advanced Science and Technology, University of Illinois at Urbana-Champaign, 405 North Mathews Avenue, Urbana, Illinois 61801, USA
[3]Department of Physics, University of Illinois at Urbana-Champaign, 1110 West Green Street, Urbana, Illinois 61801, USA
[4]Institute of Computational Comparative Medicine and Nanotechnology Innovation Center of Kansas State, Department of Anatomy and Physiology, 1800 Denison Ave, Kansas State University, Manhattan, Kansas 66506, USA
[*]jeffcomer@ksu.edu

Video legend

(`meoh_void_grid_movie.mov`) Diffusion of methanol within a pure lipid bilayer and the influence of voids on its motion. In this 60 ps fragment of a molecular dynamics simulation, the methanol molecule can be seen diffusing within a POPC membrane, with its motion affected by the spontaneous formation of empty regions (voids). These voids are shown as violet surfaces. Methanol is shown in a space-filling representation with H, C, and O atoms in white, green, and red, respectively. POPC lipids are illustrated by sticks with C, N, O, and P atoms in gray, blue, red, and tan. POPC hydrogen atoms and explicit water molecules, present in the simulation, are not shown for clarity.

Uncertainty in free energy

The error bars in Figure 1 of the main text were derived by comparing the mean gradients from samples in the first and second halves of the calculations for each window. The mean gradient over the first half, $g_s(z,T/2)$, can be extracted from the history file written by the Colvars module of NAMD,[1] while the mean gradient over only the second half can be calculated from the final mean gradient and sample count (comprising both halves):

$$g_s^{(2)}(z) = \frac{n_s(z,T)g_s(z,T) - n_s(z,T/2)g_s(z,T/2)}{n_s(z,T) - n_s(z,T/2)}, \quad (1)$$

where $n(z,t)$ and $g(z,t)$ are, respectively, the number of samples and average gradient accumulated up to time t and s is the window index. T is the total simulated time. The mean gradients from the first and second halves, $g_s^{(2)}(z)$ and $g_s^{(1)}(z) = g_s(z,T/2)$, were then combined among the windows and antisymmetrized as described in the main text. The uncertainty associated with the final gradient was taken to be half of the deviation between the gradients derived from the first and seconds halves of the simulation, i.e., $\mathrm{Err}[g(z)] = \left|g_{\mathrm{sym}}^{(2)}(z) - g_{\mathrm{sym}}^{(1)}(z)\right|/2$. As in Comer et al.,[2] the uncertainty in the gradients was propagated to the PMF. Far from the membrane, $w(z)$ was set to zero by convention. Consequently, we defined the uncertainty at the edge of the domain to be zero, $\mathrm{Err}[w(a)] = 0$, where $a = -45$ Å. As we approach the membrane, the uncertainty of $w(z)$ accumulates as we integrate over a larger stretch of uncertain gradient values. We assume that the statistical uncertainties in the gradient at each point z are independent, so we accumulate these uncertainties in quadrature by

$$\mathrm{Err}[w(z)] = \left|\int_a^z \mathrm{d}z' \, \mathrm{Err}\left[g(z')\right]^2\right|^{1/2}. \quad (2)$$

Calculating diffusivity with time-dependent biases

For the results shown in the main text, the diffusivity and fractional diffusivity was calculated from trajectories where the bias was fixed to yield a flat free-energy landscape. However, the Bayesian method was originally developed[3] for ABF calculations

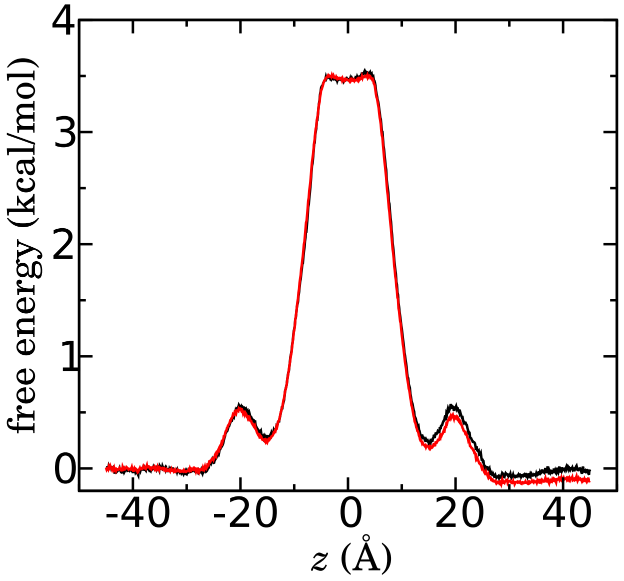

Figure S1. Convergence of the free energy calculation. Here we compare the PMF derived from the force samples in the first and second halves each simulation (combined over the 9 windows). The PMFs are anchored such that its average value on the interval $-45 < z < -43$ Å is zero. The similarity of the two PMFs provides evidence of adequate sampling.

where the bias is time dependent, eliminating the need for expending additional computational resources on simulations intended solely for the diffusivity calculation. The Bayesian scheme invoking the Smoluchowski solver presented in the main text can also be applied to simulations including time-dependent biases. In the original formulation,[3] where the solutions to the Smoluchowski equation were approximated by Gaussians, only the bias at the initial position of the diffusing particle was considered, $f^{\text{bias}}(Z(t),t)$. To be consistent with the (Crank-Nicolson) Smoluchowski solver approach, which is intended to be used for longer lag times, it is necessary to consider the function $f^{\text{bias}}(z,t)$ over the entire interval that the diffusing particle may reach in the time Δt. The complete biasing force is available in the form of history files that can be optionally written by the Colvars module.[4] In principle, computing the probability of a displacement from position $Z(t)$ at time t to the position $Z(t+\Delta t)$ at time $t+\Delta t$ would require considering changes to the biasing force during the journey, which would be extremely complex for a history-dependent bias like in ABF. Luckily, however, as the ABF calculation progresses, the biasing force changes at a decreasing rate. Once all of the bins have accumulated a large number of samples, the biasing force is unlikely to change much over an interval Δt that is significantly shorter than the simulation time. Thus, after discarding the early stages of the ABF calculations, it was possible to approximate the bias profile during a displacement by its value at the initial time. Even with this approximation, the time required for the diffusivity calculations for trajectories produced with a time-dependent bias was considerably longer than those without such a bias due to the fact that a time-dependent biasing force precludes the use of certain optimizations in the Bayesian algorithm. Note, however, the computational cost of diffusivity calculation remained miniscule compared to the molecular dynamics simulations from which the trajectories were derived. For the calculations with a fixed biasing force reported in the main text, the Smoluchowski equation only had to be solved once for each displacement in the trajectory with the same initial point during a single step of the Bayesian scheme, resulting in a large improvement in the speed of the calculation. However, this shortcut could not be used for the time-dependent bias, since each displacement takes place on a different bias profile, each yielding a distinct solutions to the Smoluchowski equation. For the classical diffusive model, it was still possible to obtain converged results for the trajectories with time-dependent biases in a reasonable time frame (less than 2 hours). The results are shown in Figure S2.

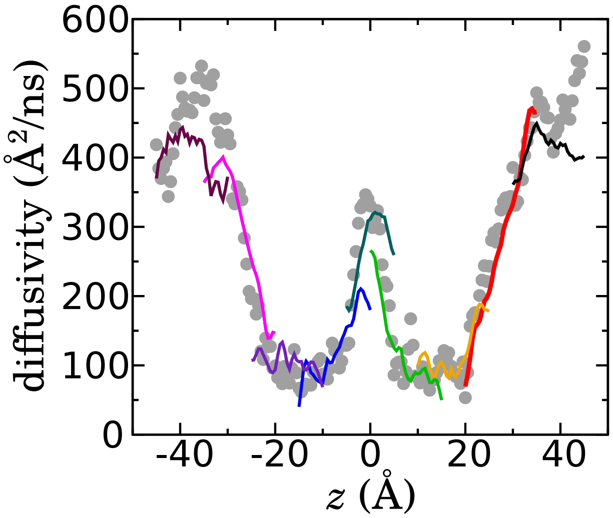

Figure S2. Diffusivity calculated from stratified ABF calculations using the Bayesian scheme and Smoluchowski solver. The gray circles show a $D(z)$ calculation on the trajectories used in the main text, i.e. 13 independent simulations where the methanol molecule freely diffused on an effectively flat free-energy landscape over the full $-45 < z < 45$ Å interval. Each colored curve represents $D(z)$ as calculated for each of the 9 windows of the ABF calculation, using the original simulation trajectories from these calculations. These stratified calculations show modest agreement with the calculation over the full interval, although some misalignment is apparent, likely owing to quasi-nonergodicity in the stratified simulations. The lag time was $\Delta t = 20$ ps for the data shown here.

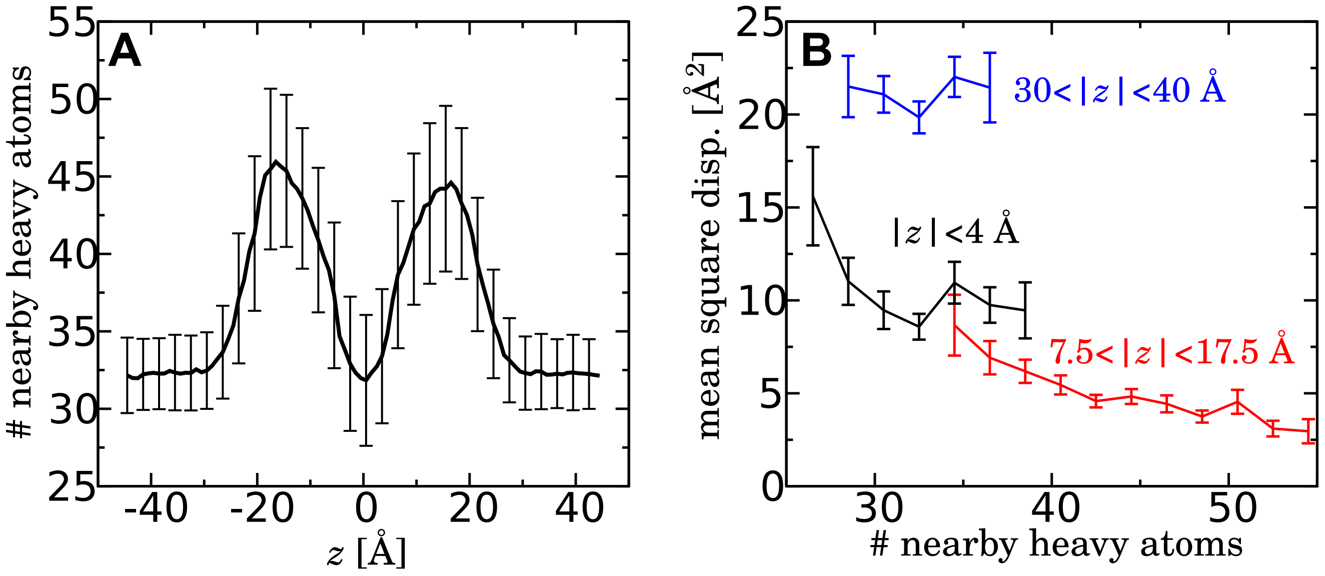

Figure S3. Effect of the local atomic density on methanol diffusion. **(A)** Number of nonhydrogen atoms within 6 Å of methanol as a function of z (position along the membrane normal). Error bars are standard deviations. Results were calculated from molecular dynamics simulations, totaling 440 ns, in which the free-energy landscape along z was made effectively flat by applying an external force to cancel the intrinsic PMF. **(B)** Mean squared displacement (MSD) of methanol as function of the number of nonhydrogen atoms within 6 Å for different regions of the membrane. Error bars are standard errors. In the aqueous phase ($30 < |z| < 40$ Å), there is little variation in the number of nearby atoms, and no significant variation in the MSD. On the other hand, within the membrane, the number of nearby atoms fluctuates over a wide range and the MSD decreases dramatically as the density of atoms increases.

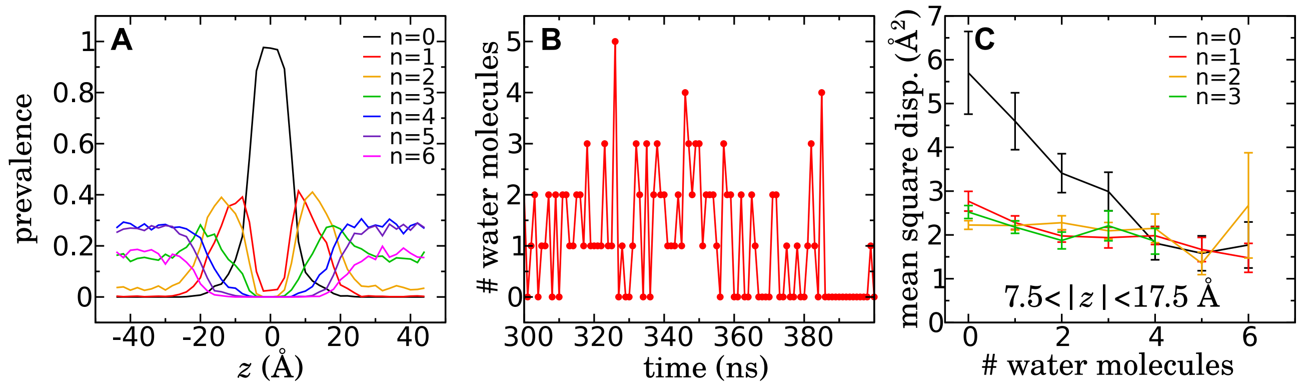

Figure S4. Water coordination of methanol during membrane permeation. **(A)** Prevalence of different water coordination numbers, n, for methanol as a function of z. Water molecules were considered to be in the first coordination shell of the alcohol when at distances < 3.5 Å, which is near the minimum of the radial distribution function in aqueous solution. Near the center of the membrane ($|z| < 6$ Å), methanol tends to be completely unsolvated. At larger distances from the center, the probability of being accompanied by one or more water molecules grows until predominated by coordination by 4 or 5 water molecules, with lower frequencies of $n = 3$ or 6, as in bulk aqueous solution. **(B)** Typical trace of water coordination for a simulation in which the methanol molecule was restrained to the interval $0 < z < 15$ Å, showing that exchange of water molecules in and out of the methanol coordination shell occurs on timescales of a few nanoseconds. **(C)** Mean square displacement of methanol in the membrane and the effect of coordinating water molecules. The largest displacments seen in Figure S3B for $7.5 < |z| < 17.5$ Å occur only when the alcohol is not coordinated by water molecules.

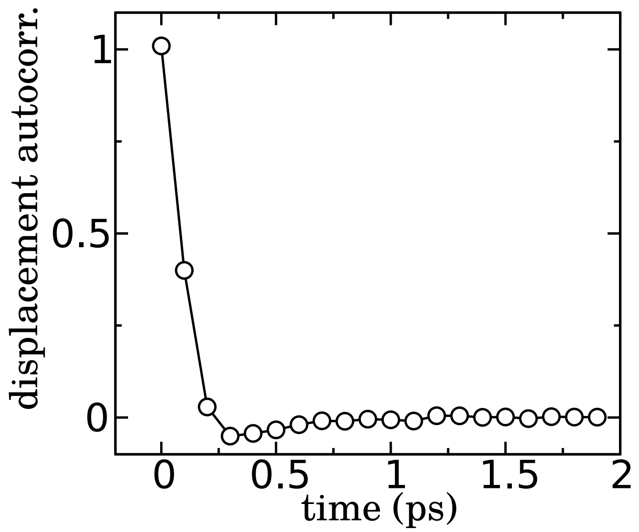

Figure S5. Autocorrelation of methanol displacements in aqueous solution. For short times (< 100 fs) motion is ballistic, with high correlation between consecutive displacements. By $t = 1$ ps, consecutive displacements are essentially uncorrelated and the motion is diffusive.

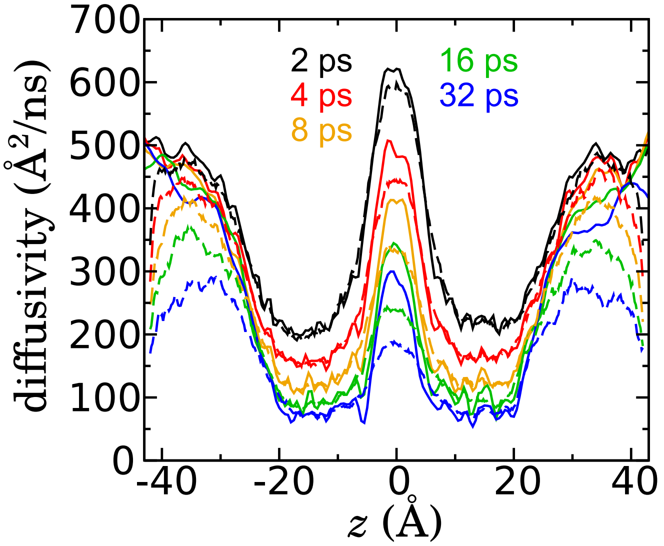

Figure S6. Comparison of the results of the Bayesian scheme using a (classical) Smoluchowski solver as presented in the main text (solid curves) and the Gaussian approximation to the Smoluchowski solutions used in previous works (dashed curves).[3,5] The latter approximation presumes that $D(z)$ changes little during the lag time Δt. Consistent with this, the Gaussian approximation works best for short Δt values and regions of low $D(z)$, both of which imply small displacements. For a lag time of $\Delta t = 2$ ps, the resulting $D(z)$ is almost identical between the two methods, except for a small artifact near the reflecting boundary in the Gaussian approximation. With increasing Δt, the accuracy of the approximation degrades in the aqueous medium, where the large $D(z)$ means that contact with the membrane becomes increasingly likely during the lag time. Loss of accuracy is also seen near the center of the membrane.

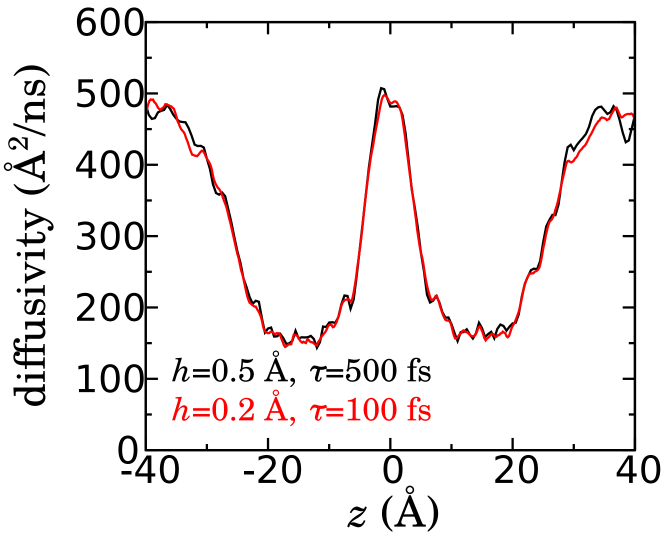

Figure S7. Comparison of the results of the Bayesian scheme using the classical Smoluchowski solver on different grids. In the main text, a spatial grid size of $h = 0.5$ Å and a time step of $\tau = 500$ fs were used. To ensure that these values were sufficiently small, we performed a calculation with a grid size of $h = 0.2$ Å, that is, 2.5 times smaller. To maintain stability, the time step should be reduced by about 2.5^2, so $\tau = 100$ fs was chosen. The results are almost identical. Here, the lag time was $\Delta t = 4$ ps.

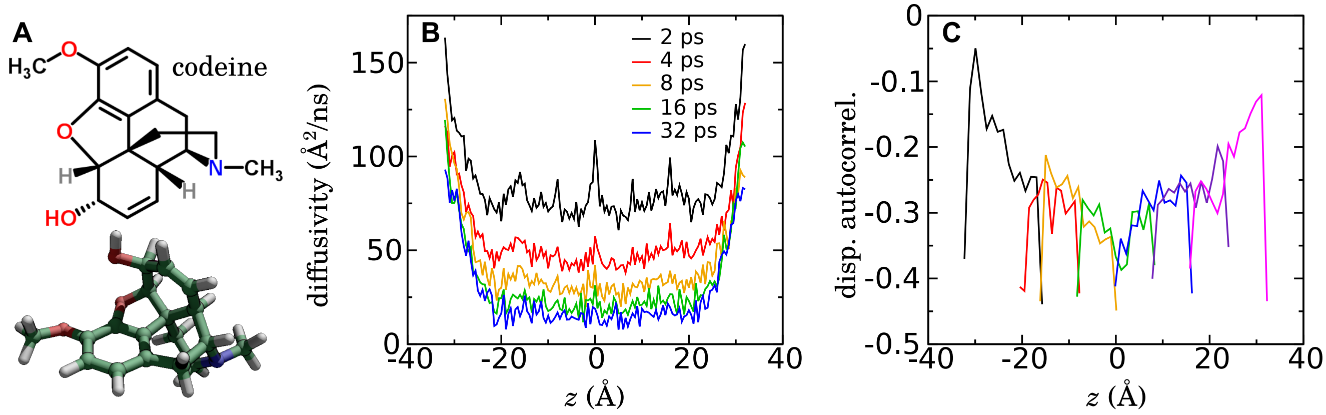

Figure S8. Evidence of subdiffusive behavior for the membrane permeation of codeine. **(A)** The structure of codeine. **(B)** $D(z)$ as calculated by the Bayesian scheme described in the text for different lag times, Δt. The predicted diffusivity $D(z)$ appears to decrease with Δt, similar to that observed for methanol in Figure 2 of the main text. **(C)** Normalized correlation of consecutive codeine displacements as a function of position z. The different colors indicate different ABF windows. As in Figure 2B of the main text, there is significant negative correlation near the center of the membrane. Large-magnitude negative correlation near the window boundaries is an artifact of the reflective boundary conditions, which was also seen at ± 45 Å in Figure 2B. The simulations from which the codeine trajectories were derived were similar to those described for methanol in the main text, except that a pure dimyristoylphosphatidylcholine (DMPC) lipid bilayer and temperature of 298.15 K were used. For further details, see Lee et al.[6]

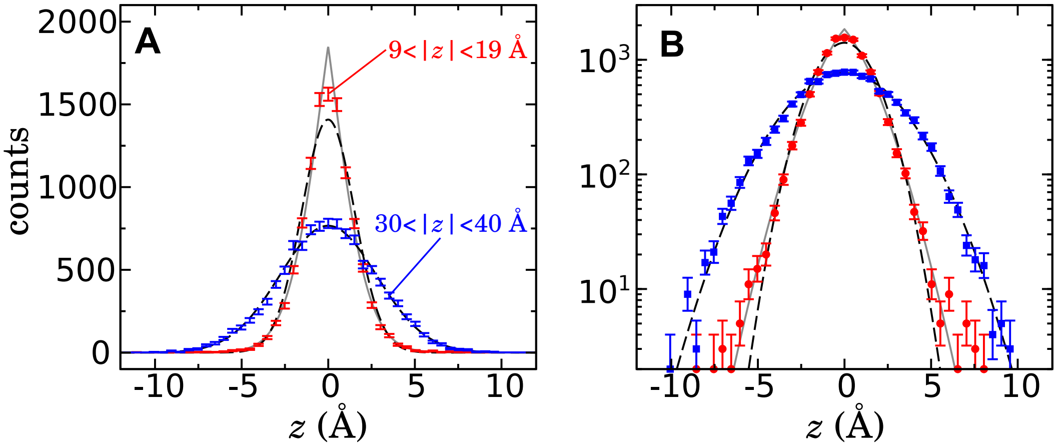

Figure S9. Probability distribution of methanol displacement along the z axis for a lag time of 10 ps on a linear **(C)** or logarithmic **(D)** scale. Dashed black lines are Gaussian curves with the same mean and variance as the probability distributions derived from the simulations, while the gray curve is a fit of the subdiffusive probability distribution given by Metzler and Klafter.[7] Lower and upper error bar magnitudes are $\sqrt{n+\frac{1}{4}}-\frac{1}{2}$ and $\sqrt{n+\frac{1}{4}}+\frac{1}{2}$, where n is the number of counts in the histogram bin.[8] This plot is similar to Figure 3 of the main text, except that, here, displacements along z are considered. The interpretation is a bit more problematic since the system is inhomogeneous along z.

Pure alkane simulations

A system of 320 hexadecane molecules was constructed using Packmol[9] and equilibrated in molecular dynamics simulation for 20 ns. The simulations were performed as described in "Molecular dynamics methods" in the main text. Atomic interactions of hexadecane were described by the CHARMM General Force Field.[10] The pressure and temperature were maintained at 1 atm and 308 K, respectively. The size of the system was approximately (54.0 Å)^3. Following equilibration, a single methanol molecule was inserted at the origin. After energy minimization, this system was simulated for 100 ns with the barostat applied to the z-axis only, which allowed diffusion of methanol along the x and y axes to be unambiguously tracked. The mean square displacement of the methanol molecule, $\langle \Delta x^2 + \Delta y^2 \rangle$, was calculated as a function of the lag time, t. Voids were identified following the same procedure detailed in "Identification of voids" in the main text. The prevalence and form of the voids was similar to that determined within the center of the lipid bilayer, as shown in Figure S10A.

The hexadecane system is in principle homogeneous when averaged over sufficiently long times, facilitating analysis of the methanol diffusion. The probability distribution of the methanol displacements for several different lag times are shown in Figure S10B,C. Fits to these curves based on the theoretical form of Metzler and Klafter,[7] (Equation 46), were also computed by maximizing $\prod_i W_\alpha(\Delta x_i, t) W_\alpha(\Delta y_i, t)$, where Δx_i and Δy_i were observed displacements of methanol. The optimal α values are plotted as a function of the lag time in Figure S10D. Another method to obtain the timescale-dependence of the fractional order α was suggested by Saxton:[11]

$$\alpha(t) = \frac{\mathrm{d} \ln \langle \Delta x^2 + \Delta y^2 \rangle}{\mathrm{d} \ln t}. \quad (3)$$

We have also plotted the results of this formula in Figure S10D. Both approaches (the W_α-fit and Saxton formula) give similar forms for $\alpha(t)$. Traces of the short-time ballistic regime may be evident in the large values of $\alpha(t)$ for $t < 1$ ps. Note that W_α-fit as implemented was incapable of giving $\alpha(t) > 1$. The Saxton formula predicts $\alpha(t) < 0.6$ for $2 \le t \le 5$ ps, while the lowest values attained by the W_α-fit approach were 0.70 to 0.74, on the interval $4 \le t \le 16$ ps. The two approaches have approximate quantitative agreement for $t \ge 16$ ps, rising gradually from 0.70 and reaching 1.0 near $t = 256$ ps.

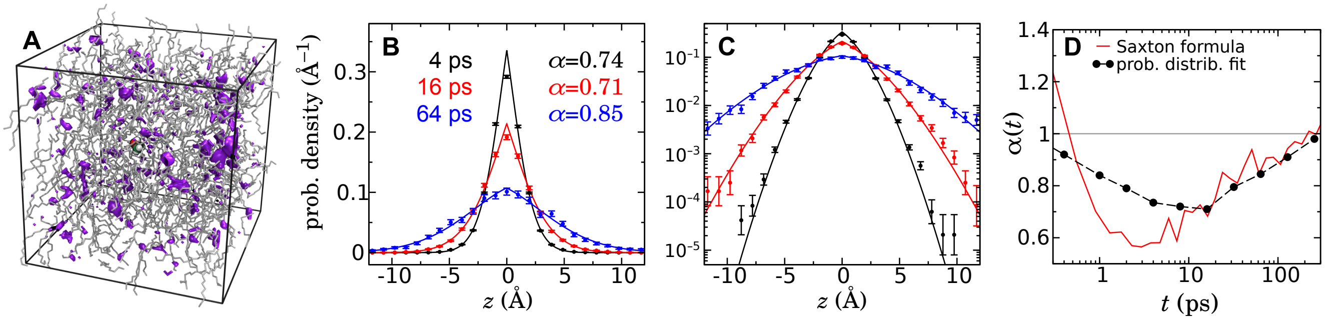

Figure S10. Simulations of methanol in hexadecane. **(A)** Snapshot of the simulation. Methanol is shown as spheres (with H, C, and O atoms colored white, green, and red, respectively). The carbon atoms of the hexadecane are shown as gray sticks; hexadecane hydrogen atoms are not illustrated. **(B)** Probability distribution of methanol displacements during the simulation for lag times of 4, 16, and 64 ps. Solid lines are fits to the theoretical curve.[7] The optimal fractional order α resulting from each fit is included on the right. **(C)** Same as panel B, but on a logarithmic scale, highlighting the behavior of the tails. **(D)** Dependence of the fractional order α on the lag time as determined by fits to the theoretical form of the probability distribution[7] and through analysis of the mean square displacement using the formula of Saxton.[11]

References

1. Phillips, J. C. *et al.* Scalable molecular dynamics with NAMD. *J. Comput. Chem.* **26**, 1781–1802 (2005).
2. Comer, J. *et al.* The adaptive biasing force method: Everything you always wanted to know but were afraid to ask. *J. Phys. Chem. B* **119**, 1129–1151 (2015).
3. Comer, J., Chipot, C. & Gonzalez-Nilo, F. D. Calculating position-dependent diffusivity in biased molecular dynamics simulations. *J. Chem. Theory Comput.* **9**, 876–882 (2013).
4. Fiorin, G., Klein, M. L. & Hénin, J. Using collective variables to drive molecular dynamics simulations. *Mol. Phys.* **111**, 3345–3362 (2013).
5. Comer, J., Schulten, K. & Chipot, C. Calculation of lipid-bilayer permeabilities using an average force. *J. Chem. Theory Comput.* **10**, 554–564 (2014).
6. Lee, C. *et al.* Simulation-based approaches for determining membrane permeability of small compounds. *J. Chem. Inf. Model.* **56**, 721–733 (2016).
7. Metzler, R. & Klafter, J. The random walk's guide to anomalous diffusion: a fractional dynamics approach. *Phys. Rep.* **339**, 1–77 (2000).
8. Heinrich, J. G. CDF/MEMO/STATISTICS/PUBLIC/6438 Version 1: Coverage of error bars for Poisson data (2003). URL http://www-cdf.fnal.gov/physics/statistics/notes/cdf6438_coverage.pdf. Accessed 2016-09-11.
9. Martínez, L., Andrade, R., Birgin, E. G. & Martínez, J. M. Packmol: A package for building initial configurations for molecular dynamics simulations. *J. Comput. Chem.* **30**, 2157–2164 (2009).
10. Vanommeslaeghe, K. *et al.* CHARMM general force field: A force field for drug-like molecules compatible with the CHARMM all-atom additive biological force fields. *J. Comput. Chem.* **31**, 671–690 (2010).
11. Saxton, M. J. Wanted: A positive control for anomalous subdiffusion. *Biophys. J.* **103**, 2411–2422 (2012).
